# Loss of Both Parotid Glands

**Published:** 1883-03

**Authors:** 


### Loss of Both Parotid Glands.

Lewis C., æt. 29, contracted chills and fever. When first seen by C. M. Ramsdell, M. D. (*Med. News*, Jan. 13, 1883), temperature was 103 degrees, which was reduced by quinine; three days afterwards his left jaw began to swell, and by noon the whole left side of neck and face was much swollen. The next day the right parotid region commenced swelling, and soon reached an enormous size. Prostration was now great. Patient was placed upon a stimulant, a pyrexial treatment, abscesses were lanced when ripe, three days from commencement of attack. They then pursued the course of an ordinary abscess until the seventh day, when

"proud flesh" was found occluding the wound. This was removed, and proved to be the parotid glands almost entire. They were of the same appearance, spongy, with deep sulci, apparently where large vessels or nerves had passed through them. The right gland retained somewhat its natural shape, but the left could be determined alone by its internal structure. No other glands were implicated, nor any burrowing of pus. A month sufficed to complete the cure. Patient complained of having more "heart-burn" than he used to have. His mouth seemed dryer than natural. Steno's duct was closed on the left side; on the right it consisted of a *cul-de-sac* about a fourth of an inch deep. The remaining salivary glands have taken an increased functional activity, and the above conditions are ameliorated.

"This case seems to be unique. Flint says that parotiditis is an occasional complication of typhus and typhoid, and in such cases considerable sloughing of the areolar tissue frequently occurs; but neither he nor any other writer, so far as I know, mentions loss of the gland as a possible result."

